# The complete mitochondrial genome sequence of spinach, *Spinacia oleracea* L

**DOI:** 10.1080/23802359.2017.1334518

**Published:** 2017-06-01

**Authors:** Xiaofeng Cai, Chen Jiao, Honghe Sun, Xiaoli Wang, Chenxi Xu, Zhangjun Fei, Quanhua Wang

**Affiliations:** a Development and Collaborative Innovation Center of Plant Germplasm Resources, College of Life and Environmental Sciences, Shanghai Normal University, Shanghai, China;; b Boyce Thompson Institute, Cornell University, Ithaca, NY, USA

**Keywords:** Spinach, mitochondrial genome, next generation sequencing

## Abstract

Spinach *(Spinacia oleracea* L.) is an economically important vegetable crop. Here we describe the complete mitochondrial DNA sequence of spinach, which has a length of 329,613 bp and a GC content of 43.4%. It is separated by a pair of directly repeated elements of 7286 bp, to form a large single copy region of 229,375 bp and a small single copy region of 85,666 bp. The genome contains 29 protein-coding genes, 4 pseudogenes, 24 tRNA genes, and 3 rRNA genes. A phylogenetic analysis revealed that spinach was closely related to *Beta vulgaris* (sugar beet), both belonging to the Amaranthaceae family.

Spinach *(Spinacia oleracea* L.) is widely cultivated as an economically important green leafy vegetable crop for fresh consumption and processing (van Treuren et al. 2011), and is a rich source of several important health-promoting nutrients including carotenoids, folates and vitamin C. Spinach is a member of the Amaranthaceae family, which includes several other important crops such as beets and quinoa. Spinach is native to central Asia and thought to have originated in Persia (Iran), and the genus *Spinacia* contains two wild species, *S. turkestanica* and *S. tetrandra* (Ryder 1979). However, no complete mitochondrial genome sequences are currently available for *Spinacia* species.

In this study, we determined the complete mitochondrial genome sequence of *S. oleracea*. Genomic DNA was extracted from young leaves of an inbred line (SP75) that was collected from Development and Collaborative Innovation Center of Plant Germplasm Resources at Shanghai Normal University campus (30°50′29.4”N, 121°31′16.7”E), and used to construct Illumina DNA libraries. High-throughput sequencing generated ∼169 Gb high-quality cleaned sequences, which were used previously to assemble a draft nuclear genome (Xu et al. 2017). Meantime, these sequences were also used for *de novo* assembly using SOAPdenovo2 (Luo et al. 2012) and the final assembled contig that was from *S. oleracea* mitochondrion was identified based on the k-mer depth analysis and the comparison to other plant mitogenome sequences. The mitochondrial genome of *S. oleracea* (GenBank accession number KY768855) is a typical circular DNA molecule with 329,613 bp in length. It includes a pair of directly repeated elements with a length of 7286 bp. The directly repeated elements separate the genome into a large single copy region of 229,375 bp and a small single copy region of 85,666 bp. The structure of the *S. oleracea* mitochondrial genome is consistent with that reported before, which was determined based on the physical mapping of mitochondrial DNA (Stern & Palmer 1986). The overall GC content of the genome is 43.4%, and the GC content in exons is slightly lower (41.8%). The genome contains 29 protein-coding genes and 4 pseudogenes, of which 6 have multiple exons. Two of the multi-exon genes, nad2 and nad5, were trans-spliced in the mitochondrial genome. The total number of exons in the genome is 44, with a total size of 27,708 bp (about 8.4% of the total mitogenome) and an average length of 630 bp. In addition, the genome contains 24 tRNA genes coding for 16 amino acids, and 3 rRNA genes including a 5S rRNA, an 18S rRNA, and a 26S rRNA.

A neighbour-joining phylogenetic tree of *S. oleracea* and 12 other representative plant species was constructed with single-copy orthologous genes in the mitogenomes using MEGA6 (Tamura et al. 2013). As shown in the phylogenetic tree, nearly all nodes were strongly supported with high bootstrap values (Figure 1). The result suggested that as expected, *S. oleracea* was placed closely to *Beta vulgaris* (sugar beet), both of which belong to the Amaranthaceae family of the order of Caryophyllales, which constitutes the basal clade in core eudicots (Dohm et al. 2014).

**Figure 1.**
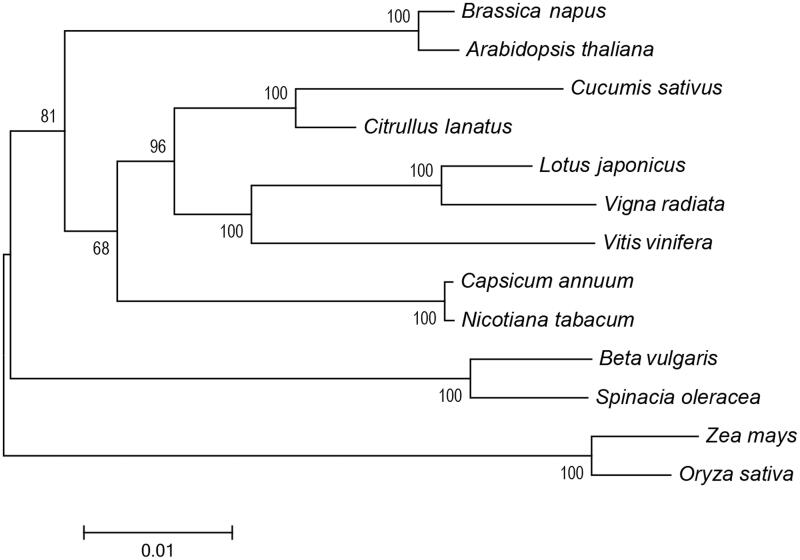
Neighbour-joining phylogenetic tree of *Spinacia oleracea* and 12 other plant species based on the single-copy orthologous genes in the mitochondrial genomes. GenBank accession numbers: *Arabidopsis thaliana* (NC_001284); *Beta vulgaris* (NC_015099); *Brassica napus* (NC_008285); *Capsicum annuum* (NC_024624); *Citrullus lanatus* (GQ856147); *Cucumis sativus* (NC_016005); *Lotus japonicus* (NC_016743); *Nicotiana tabacum* (NC_006581); *Vigna radiata* (NC_015121); *Vitis vinifera* (NC_012119); *Oryza sativa* (DQ167399); *Zea mays* (NC_007982).
